# DNA-free CRISPR genome editing in raspberry (*Rubus idaeus*) protoplast through RNP-mediated transfection

**DOI:** 10.3389/fgeed.2025.1589431

**Published:** 2025-06-30

**Authors:** Ryan Creeth, Andrew Thompson, Zoltan Kevei

**Affiliations:** Centre for Soil, Agrifood and Biosciences, Cranfield University, Cranfield, United Kingdom

**Keywords:** CRISPR-Cas9, genome editing, Rubus idaeus, DNA-free, RNPs, protoplasts

## Abstract

Protoplast-based systems have been utilised in a wide variety of plant species to enable genome editing without chromosomal introgression of foreign DNA into plant genomes. DNA-free genome editing followed by protoplast regeneration allows elite cultivars to be edited without further genetic segregation, preserving their unique genetic composition and their regulatory status as non-transgenic. However, protoplast isolation presents a barrier to the development of advanced breeding technologies in raspberry and no protocol has been published for DNA-free genome editing in the species. Pre-assembled ribonucleoprotein complexes (RNPs) do not require cellular processing and the commercial availability of Cas9 proteins and synthetic guide RNAs has streamlined genome editing protocols. This study presents a novel high-yielding protoplast isolation protocol from raspberry stem cultures and RNP-mediated transfection of protoplast with CRISPR-Cas9. Targeted mutagenesis of the phytoene desaturase gene at two intragenic loci resulted in an editing efficiency of 19%, though estimated efficiency varied depending on the indel analysis technique. Only amplicon sequencing was sensitive enough to confirm genome editing in a low efficiency sample. To our knowledge, this study constitutes the first use of DNA-free genome editing in raspberry protoplast. This protocol provides a valuable platform for understanding gene function and facilitates the future development of precision breeding in this important soft fruit crop.

## Introduction

Raspberry (*Rubus idaeus*; RRID:NCBITaxon_32247) is a high-value horticultural crop that, alongside other *Rubus* berries, has undergone a substantial increase in consumption worldwide in recent decades (Foster et al., 2019). Globally, production increased 48% between 2011 and 2021 (FAO, 2024). Often sold as a “superfood,” raspberries contain high levels of anthocyanins, flavanols and phenolic acids that are essential for normal metabolism. Lack of dietary sources of these bioactive nutrients has been associated with cancer, stroke, Alzheimer’s and autoimmune diseases (Derrick et al., 2021; Hancock et al., 2007). Raspberry is a highly heterozygous species that is not true to seed, meaning both sexual reproduction and selfing can introduce allelic variation that alters the berry phenotype of elite cultivars. Commercial raspberry production thus relies on vegetative propagation. This challenge, in addition to a 2-year fruiting cycle in floricanes, has contributed to a lack of progress on next-generation methods for raspberry breeding.

Improving agricultural sustainability is a key priority for reaching net zero (FAO. Emissions due to agriculture, 2020). Food production systems must undergo sustainable intensification, and gains can be made in raspberry production through improving efficiency while reducing food waste. Advanced breeding to improve traits such as fungal resistance or fruit firmness would substantially improve shelf life (Simpson et al., 2017). Enhanced plant and fruiting architecture could reduce labour demands for crop management and harvesting while eliminating genetic disorders, such as crumbly fruit syndrome, would prevent widespread yield losses (Scolari et al., 2023). Such improvements can also minimise chemical inputs and decrease overall land use, leading to more productive and sustainable raspberry cultivation. Greater access to this nutritious species would also support the UN’s 2030 Sustainable Development Goals of zero hunger and good health and wellbeing (United Nations, 2024).

The advent of CRISPR (clustered regularly interspaced short palindromic repeats) genome editing has greatly increased the scope and speed of plant breeding (Jinek et al., 1979). Through the formation of ribonucleoprotein complexes (RNPs), composed of Cas nucleases and programmable guide RNA (gRNA), targeted mutagenesis of any genomic region of interest can be achieved (Gasiunas et al., 2012; Sternberg and Doudna, 2015; Wright et al., 2016). The most commonly used nuclease, Cas9, induces a double strand break (DSB) in genomic DNA at a specific locus identified by complementary binding of the gRNA (Zhan et al., 2021). Often, error-prone DNA repair subsequently creates insertions and/or deletions (indels) at the specified locus, leading to gene knockout (KO).

Applying this system to plant species is challenging as the plant cell wall is impermeable to nucleic acids encoding Cas9/gRNA or pre-assembled RNPs. Inbreeding, annual, seed-propagated crops can utilize *Agrobacterium*-mediated transformation or biolistics to transfer DNA encoding CRISPR/Cas9 into intact cells and to integrate this into chromosomes. After genome editing has occurred, transgenes can be easily removed by backcrossing and genetic segregation as transgenic crops have historically been contentious from regulatory and public perception standpoints (Chan et al., 2020; Turnbull et al., 2021). However, to realise the benefits of genome editing in the advanced breeding of allogamous, clonally-propagated crops, such as raspberry, editing must take place without chromosomal introgression of foreign DNA. This allows elite clones to be edited without the need for further genetic segregation, preserving their unique genetic composition and their regulatory status as non-transgenic. Where *Agrobacterium*-mediated transformation cannot be used, the resurgence of protoplast-based techniques offers a transgene-free alternative (Yue et al., 2021; Yoo et al., 2007; Barceló et al., 2019; Reed and Bargmann, 2021; Hsu et al., 2021; Woo et al., 2015).

Enzymatic digestion of the plant cell wall (enzymolysis) liberates single-celled protoplasts (Yoo et al., 2007; Cocking, 1960; Davey et al., 2005) with membranes permeable to plasmids and pre-assembled RNPs in the presence of polyethylene glycol (PEG) (Woo et al., 2015). After editing, protoplast can be regenerated into whole plants (Reed and Bargmann, 2021; Jeong et al., 2021; Scintilla et al., 2022), although this is technically challenging and species dependent. Cas9 and gRNA genes encoded within plasmids are expressed within the protoplast nucleus, triggering genome editing, but do not integrate into the genome. Pre-assembled RNPs, exogenously synthesised and formed extracellularly, are also able to directly translocate the protoplast membrane to initiate editing (Woo et al., 2015). RNPs are then degraded by normal cellular processes. This form of DNA-free genome editing is rapid, highly specific and indistinguishable from natural mutagenesis–factors that have recently found favour with regulators (Genetic Technology (Precision Breeding) Act, 2023; European Commission, 2021). Pre-assembled RNPs are of particular interest in crop species like raspberry as they eliminate the chance of random integration of DNA into the genome, which is a risk with plasmid transfection. The commercial availability of synthetic gRNAs and Cas nucleases from many vendors, and evidence for higher editing efficiencies than plasmid-based techniques (Zhang Y. et al., 2022), also favour the use of DNA-free RNPs.

Genome editing and genetic engineering in raspberry have not been extensively developed (Foster et al., 2019). *Agrobacterium*-mediated transformation has been achieved by several groups (Mathews et al., 1995; Mezzetti et al., 2004; Kim and Dai, 2022; Zhang J. et al., 2022; Khadgi, 2020), however transgenic raspberries have never been commercialised. To our knowledge, no method for DNA-free CRISPR genome editing in raspberry has been published, however there have been attempts to use CRISPR transgenically (Khadgi, 2020; Miller, 2019), with some potential success with biolistic delivery. Nonetheless, there is a substantial research gap in this field. Pre-assembled RNPs present an excellent opportunity to rapidly test gRNAs if reliable and modern protoplast-based methods can be elucidated. Existing protoplast research on raspberry is limited in scope and predates the advent of genome editing (Phan et al., 1997; Nita-Lazar et al., 2000; Mezzetti et al., 1999), but provides evidence that raspberry protoplasts can be isolated and cultured. A high-yielding tissue source of protoplast is critical, but high heterozygosity in raspberry complicates the issue as tissue-cultured seedlings, used in many other protocols [e.g. (Yoo et al., 2007)], would result in allele segregation and genetically variable regenerated plants and compromise the elite berry phenotype of commercial raspberry cultivars. Nonetheless, many raspberry cultivars are suitable for tissue culture; vegetative propagation is common industry practice (Funt and Hall, 2013) and raspberry produces shoots vigorously, including directly from roots, suggesting a propensity for regeneration. As there is no selection in protoplast regeneration, reporter genes such as *phytoene desaturase* (*PDS*) are useful to assess the success of genome editing in regenerated plants, particularly during initial development of protoplast regeneration. KO of *PDS*, which plays a critical role in carotenoid biosynthesis, results in a distinctive albino phenotype that is visible during the early stages of regeneration (Lin et al., 2018). Functional targets are also of interest, particularly those that could be beneficial to raspberry shelf life and where gene KO results in an improved phenotype. For example, evidence from other fruit species indicates that *polygalacturonase* (*PG*) can increase fruit firmness (López-Casado et al., 2023; Atkinson et al., 2012; Posé et al., 2013), *WRKY52* can improve resistance to *B. cinerea* (Jia et al., 2020) and powdery mildew (Wang et al., 2017), and *nonexpressor of pathogenesis-related genes 1* (*NPR1*) can also improve resistance to *B. cinerea* (Li et al., 2020; Li et al., 2021).

This study demonstrates successful, DNA-free genome editing of raspberry protoplasts using CRISPR-Cas9 for the first time. We have applied state-of-the-art protoplast methods to a new species and achieved a high genome editing efficiency. These are critical steps in the development of next-generation breeding technologies (NGBTs) in raspberry and will hopefully stimulate further work in an underserved yet economically and nutritionally valuable species.

## Materials and methods

### Plant cultivation

Cold-treated (4°C, ≥50 days, ∼15 × 20 cm) roots of *R. idaeus* cultivar BWP102 (supplied by a commercial propagator) were planted in 7.5 L pots containing peat-rich soil (Sinclair All Purpose Growing Medium) with perlite and fertilised with Hoagland’s solution (Hoagland and Arnon, 1950) twice a week. Highly vigorous, 1.5 cm diameter, 1 m tall canes produced from cold-treated roots were cut down after approximately 1 month in a partially environmentally controlled glasshouse (set points of 20°C, 16:8 hrs day: night, supplemented with high-pressure sodium lamps). Petioles and leaves were removed, and cane stems cut into 50–70 mm segments containing one axillary bud. Cuttings were sterilised in 70% v/v ethanol for 30 s and 10% v/v sodium hypochlorite (with 0.002% Tween 20) for 4 minutes (Funt and Hall, 2013). Exposed stem ends were cut off and stem explants were planted in Murashige and Skoog (MS) basal media with Gamborg B5 vitamins (Melford; pH 5.8, 30 g L^-1^ sucrose, 7.5 g L^-1^ agar, 1 mg L^-1^ 6-benzyl-aminopurine, 0.1 mg L^-1^ indole-3-butyric acid) within autoclaved Magenta™ boxes (Merck).

Tissue culture material was grown in a Sanyo MLR-350 growth cabinet with 45 μmol m^-2^ s ^-1^ light from fluorescent tubes (Toshiba FL40SSW/37) at 25°C with a 16: 8h day: night cycle.

### Protoplast isolation

Protoplasts were isolated from plantlets (stem cultures) grown from the axial bud cane cuttings. This material was chosen as a protoplast source as it is vegetatively grown, sterile, soft and young; therefore, it is amenable to enzymatic digestion. Plantlets (0.3 g, 20–30 mm tall) from axillary buds were harvested after 10 days of culture and cut with a sterile scalpel blade into 0.5–1 mm strips, then immediately immersed in 13 mL enzymolysis solution (20 mM MES pH 5.8, 0.4 M mannitol, 20 mM KCl, 10 mM CaCl_2_, 1.5% Cellulase R-10 (Duchefa), 0.5% Macerozyme R-10 (Duchefa)) in a 90 mm Petri dish (Yoo et al., 2007). The tissue was vacuum infiltrated for 30 min and then incubated on an orbital shaker at 50 rpm for 16 h in the dark at 28°C. Protoplast were harvested by adding 10 mL of W5 media (2 mM MES pH 5.8, 154 mM NaCl, 125 mM CaCl_2_, 5 mM KCl) to the Petri dish and agitating gently, then filtering the suspension through a 70 µM cellulose filter (Fisher Scientific) into a 50 mL centrifuge tube. Protoplast were pelleted by centrifugation (Sigma 3-16 KL) at 200 rcf for 4 minutes with slow centrifugal acceleration and breaking (both set at level 6). The supernatant was removed, and protoplasts were resuspended in W5 for a second wash at 200 rcf for 4 minutes. Finally, the supernatant was removed, and 2 mL 0.4 M mannitol (pH 5.8) was added to resuspend the protoplast.

Protoplast were then purified through a sucrose cushion (Jeong et al., 2021): 6 mL of 0.6 M sucrose solution was gently overlaid with the 2 mL protoplast-mannitol suspension in a 15 mL centrifuge tube and centrifuged at 80 rcf for 5 minutes. Protoplast suspended at the interface were carefully aspirated into a new 15 mL tube and then centrifuged at 200 rcf for 4 minutes and resuspended in 5 mL MMG (4 mM MES pH 5.8, 0.4 M mannitol, 15 mM MgCl_2_) solution to form a highly concentrated pellet of viable protoplast. Sucrose purification was only successful when initial protoplast yields exceeded 1 × 10^6^ protoplast mL^-1^.

### *In vitro* cleavage test and gRNA design

PCR product of BWP102 *phytoene desaturase* (*PDS*) was used to test the efficacy of two synthetic gRNAs that targeted separate regions of the gene *in vitro*. *PDS* in raspberry was identified by BLAST mapping strawberry (*Fragaria vesca*) *PDS* sequence to a novel genome assembly of raspberry cultivar BWP102 (Kevei et al., unpub.) in Geneious Prime v.2024.0.7 (Dotmatics, RRID:SCR_010519). gRNAs and primers were designed using the same genome assembly to amplify two separate coding regions of *PDS* (*PDS1, PDS2*) with gRNA cut sites producing uneven cleavage products distinguishable by gel electrophoresis (Table 1). gRNA1 and gRNA2 targeted separate loci in exon 13 and eight of *PDS* respectively. Custom Invitrogen TrueGuide gRNAs and Invitrogen TrueCut Cas9 were commercially synthesised by Thermo Fisher Scientific. Additional, functionally relevant gRNAs and primers were also designed targeting *PG*, *WRKY52* and *NPR1* and tested through *in vitro* cleavage tests (Supplementary Table S1).

**TABLE 1:** Oligonucleotides and gRNAs used in this study. Predicted cleavage products were deduced assuming DSB 3bp upstream from PAM site. NGS primer pairs flank the same respective gRNA binding site.

Oligo feature	*PDS* CRISPR site 1 (*PDS1 -* gRNA1 - RNP1)	*PDS* CRISPR site 2 (*PDS2 -* gRNA2 - RNP2)
Forward Primer (5′ to 3′)	AAA​ATA​AAA​TTG​AGA​ACT​ATG​CCC​TGG	CAT​GCC​TTC​ATG​TGC​ATC​ATT
Reverse Primer (5′ to 3′)	ATA​TAT​GGA​AAA​GCT​CAA​TGG​TAC​CG	AGG​CAC​TAG​GCG​TTT​GAA​GA
gRNA sequence (5′ to 3′)	GCU​AAA​UAG​AAA​CCC​UCC​AAG​GG	GAU​CAU​AUC​CAG​UCA​UUG​GG
Amplicon size (bp)	497	593
gRNA binding site within amplicon (bp)	213	346
Predicted cleavage products (bp)	285 + 212	346 + 247
NGS Forward Primer (5′ to 3′)	TTA​GAC​TGC​TGC​TCG​ACG​TG	CCC​TGA​GAG​ACT​CTG​TTC​GC
NGS Reverse Primer (5′ to 3′)	CAA​TCG​CCT​GTG​CAC​AAA​GT	TCA​GGC​ACT​AGG​CGT​TTG​AA
NGS Amplicon Size (bp)	241	294

*PDS*, phytoene desaturase; gRNA, guide RNA; RNP, ribonucleoprotein complex; bp, basepair; NGS, next-generation sequencing.

DNA was extracted from BWP102 leaf tissue with E.Z.N.A Plant DNA kit (Omega Bio-tek) and amplified with Phusion high-fidelity polymerase (Thermo Fisher Scientific), followed by purification with QIAquick PCR purification kit (QIAGEN). The *in vitro* cleavage test was followed as in Brandt et al. (2020) (Brandt et al., 2020). In brief, 1 µg gRNA and 1 µg Cas9 were pre-mixed and incubated with 200 ng PCR product for 2 hours. The sample was run on a 2% agarose gel (0.5% Tris Borate EDTA buffer, 0.005% Safeview (NBS Biologicals)). RNP activity was identified by the cleavage of the PCR product into two distinct smaller bands of expected sizes.

### Protoplast transfection

The protoplast suspension was centrifuged at 200 rcf for 4 minutes with low acceleration and deceleration after sucrose cushion filtration. The supernatant was removed as much as possible; 0.05% fluorescein diacetate (FDA) was added to a 10 µL subsample and protoplast yield was determined via haemocytometry on a fluorescent microscope (Leica Microsystems, DM2500, RRID:SCR_020224). Protoplast concentration was standardised to 1 × 10^6^ cells mL^-1^ in MMG and 200 µL (2 × 10^5^ protoplast total) was transferred to a sterile 1.5 mL microcentrifuge tube using wide-bore pipette tips.

RNPs were formed by mixing 10 µg of gRNA, 10 µg Cas9, 2 µL lipofectamine CRISPRMAX (Thermo Fisher Scientific), 2 µL Plus reagent, 2 µL NEB 3.1 buffer (New England Biolabs) up to 20 µL with ultrapure H_2_O in a PCR tube and incubated for 10 min at 25°C. Negative controls were identical but excluded gRNA. RNPs were added to the protoplast suspension followed immediately by 220 µL 40% PEG4000 (Merck) solution (0.2 M mannitol, 0.1 M CaCl_2_, 2 g PEG4000, up to 5 mL ddH_2_O) (Scintilla et al., 2022; Wang et al., 2022; González et al., 2020; Gou et al., 2020; Lee et al., 2024). The RNP-protoplast suspension was pipetted 5–10 times with a wide-bore tip and incubated for 10 min (Scintilla et al., 2022; Lee et al., 2024; Wu et al., 2017) at 25°C. Transfection was terminated by the addition of 800 µL of W5 and the suspension transferred to a clean 15 mL tube and centrifuged for 2 minutes at 200 rcf. The supernatant was discarded and 800 µL W5 added for a second wash through centrifugation for 2 minutes at 200 rcf. The supernatant was removed and 500 µL of basic culture media (MS pH 5.8, 0.4 M mannitol, 30 g L^-1^ sucrose) was added to resuspend the protoplast and the suspension was left for 24 h (Park et al., 2019). Protoplast were lysed by centrifugation at 10,000 rcf for 1 minute, the culture media was aspirated, and DNA was then extracted with the E.Z.N.A Plant DNA kit (Omega Bio-tek).

### Determination of editing efficiency with T7EI and deconvolution

Editing efficiency was determined both *in vitro* and *in silico* for PDS samples. Protoplast DNA was amplified by PCR with Phusion polymerase with the same respective primer pairs used for the *in vitro* cleavage test. Amplification was visualised on a 2% agarose gel. PCR products were purified with QIAquick PCR purification kit and the T7 endonuclease I (T7EI) digestion protocol was followed (New England Biolabs, 2023) with minor modifications. In brief, PCR amplicons were denatured at 98°C for 30 s and then annealed by decreasing the temperature gradually by −2°C s^-1^–85°C, then −0.1°C s^-1^–25°C. T7EI (New England Biolabs) was added and incubated for 1 hour at 37°C (Brandt et al., 2020). Annealed amplicons were purified again and then run on a 2% agarose gel for 45 min. PCR product from the same genome edited protoplast DNA samples was purified and sent to GENEWIZ (Azenta Life Sciences) for Sanger sequencing. Files from negative control and experimental samples were uploaded onto Tracking of Indels by Decomposition (TIDE) and deconvoluted (Brinkman et al., 2014) to estimate editing efficiency.

### Determination of editing efficiency with NGS

PCR product from the same *PDS* genome edited protoplast DNA samples described for T7EI/deconvolution and for *PG, WRKY52* and *NPR1* was purified and also sent to GENEWIZ (Azenta Life Sciences) for short-read Illumina next-generation sequencing (NGS; Amplicon-EZ). Different primer pairs flanking the same gRNA binding sites were used for *PDS* NGS as Amplicon-EZ requires primer pairs <450 bp (Table 1). FASTQ. files from the amplicon sequencing (≤70k sequences) were paired, trimmed and merged. Total wild-type and variant sequence percentages were compared to identify CRISPR indels in Geneious Prime (Prime, 2024).

## Results

### Tissue culture and protoplast isolation

**FIGURE 1:**
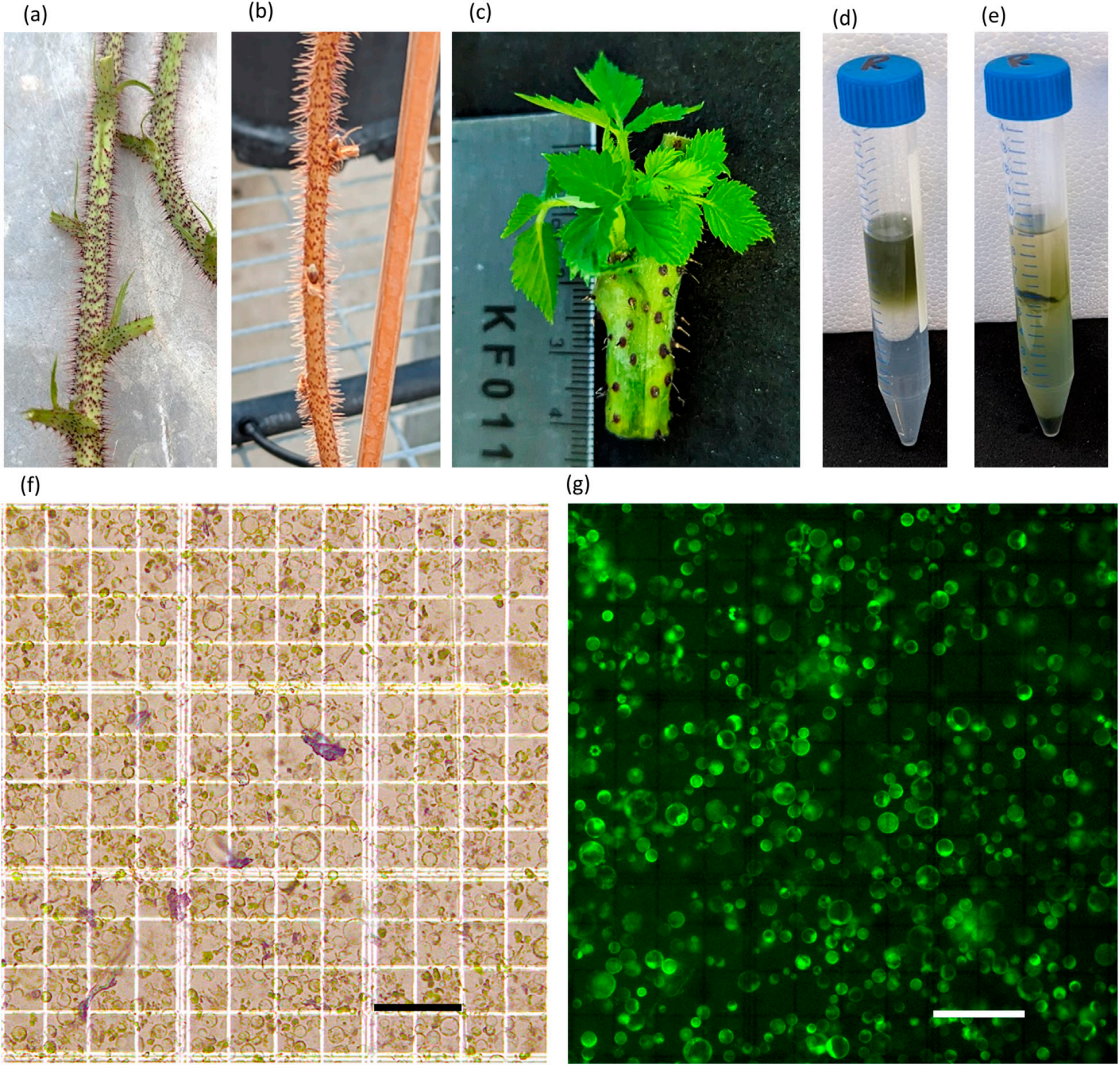
Stages of protoplast isolation in raspberry. **(A)** High-quality canes used for shoot culture, **(B)** low-quality cane, **(C)** young plantlets formed from shoot cultures, **(D,E)** purification of protoplast before **(D)** and after **(E)** sucrose cushion, **(F,G)** protoplast isolated from plantlets under bright-field **(F)** and fluorescent **(G)** illumination (bars = 100 µM).

The quality of the initial raspberry canes was a critical factor in protoplast isolation (Figures 1A,B). Cuttings from highly vigorous, 1-month old canes with no signs of senescence (no wilting/discolouring of any leaves, deep red thorns, thick bright green stem) generated fast growing plantlets (Figure 1C) that yielded between 1 × 10^6^ to 1.2 × 10^7^ protoplasts ml^-1^, (3.3 × 10^6^ g^-1^ to 3.6 × 10^7^ g^-1^) with an average of ∼5 × 10^6^ protoplast ml^-1^ (1.5 × 10^7^ g^-1^) (Figures 1F,G).

Protoplast isolated from plantlets were 10–40 µM in diameter and bright green in colour. The sucrose cushion allowed the concentration of viable protoplast and removal of debris and dead cells (Figures 1D,E). FDA staining demonstrated that the vast majority of protoplast were viable for transfection.

### *In vitro* cleavage of DNA amplicons by RNPs

**FIGURE 2:**
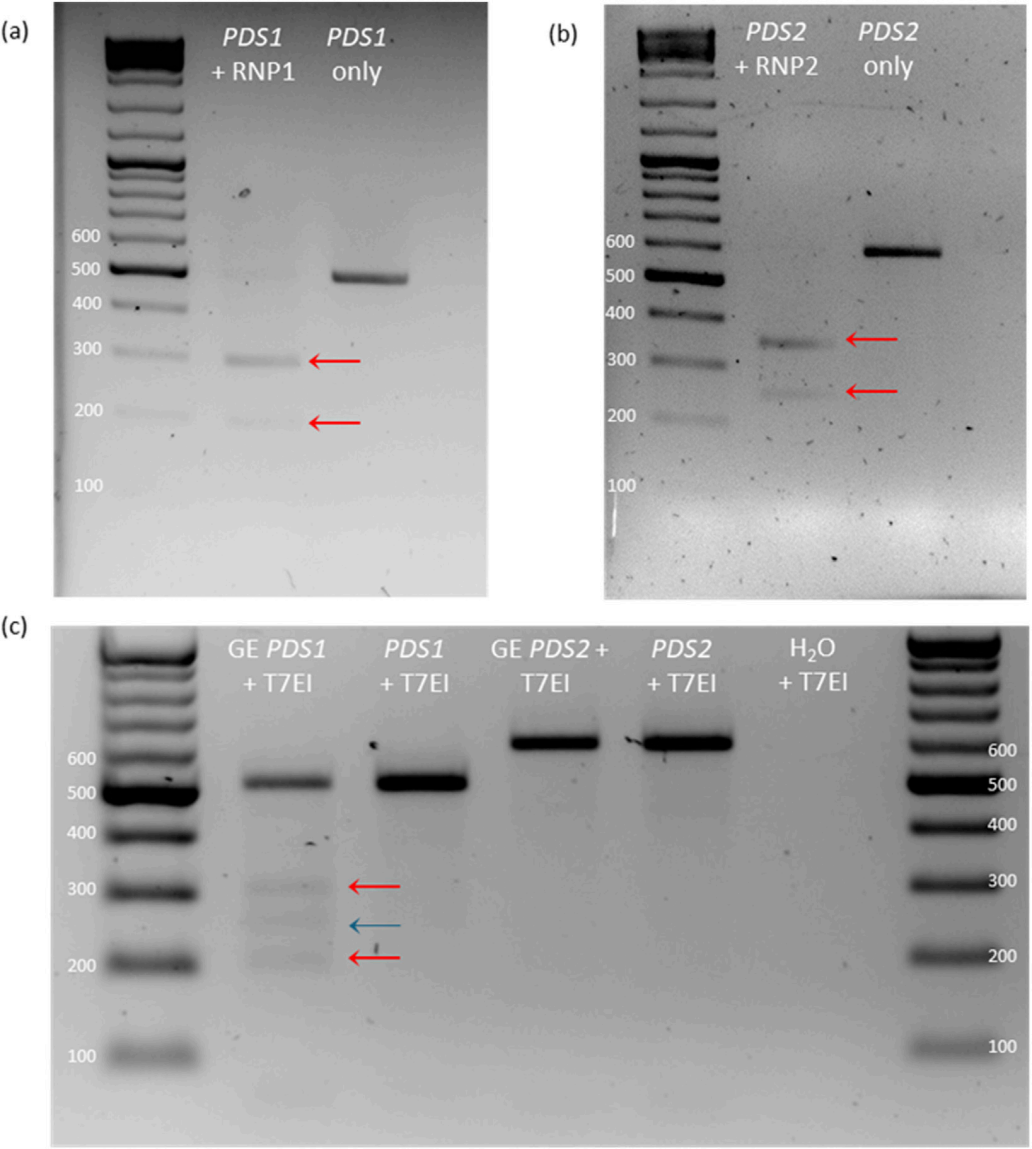
Detection of *in vitro* RNP activity and genome editing in protoplasts. **(A,B)** *In vitro* cleavage test of RNP1 **(A)** and RNP2 **(B)** on leaf-derived *PDS* PCR product. **(C)** T7EI assay of *PDS* PCR product of gene edited (GE) protoplast transfected with RNP1 and RNP2. Red arrows indicate predicted cleavage products, and the blue line indicates the additional cleavage product.

Both *PDS* RNPs showed a high level of activity against PCR amplicons with two bands clearly visible below the original band (Figures 2A,B). Compared to negative controls, the original PCR product was much reduced in intensity–indicating almost complete cleavage of the amplicons. *PDS* RNP1 and RNP2 cleavage resulted in predicted bands at approximately 212 bp + 285 bp and 247 bp + 346 bp, respectively. Cleavage by the RNPs was highly specific as no other bands were visible (Figures 2A,B). Successful *in vitro* cleavage was also seen in amplicons of *PG*, *WRKY52* and *NPR1* by their respective RNPs (Supplementary Figure S1).

### Detection of genome editing in protoplasts

The T7EI assay confirmed the presence of mutations in the *PDS1* sample through the presence of cleavage products beneath the WT amplicon band. The T7 endonuclease cleaves mismatched DNA, therefore degrading and gradually reannealing a mixed pool of protoplast-derived WT and edited amplicons results in mismatched double-stranded DNA. One strand contains indels produced through genome editing, and the other strand contains the WT sequence, creating a cleavable mismatch. The two bands were approximately the predicted sizes at 212 bp and 285 bp, however there was an additional band at 250 bp. Furthermore, the WT amplicon band was also higher than expected at approximately 510 bp. However, *PDS2* showed no cleavage by T7EI (Figure 2C).

**FIGURE 3:**
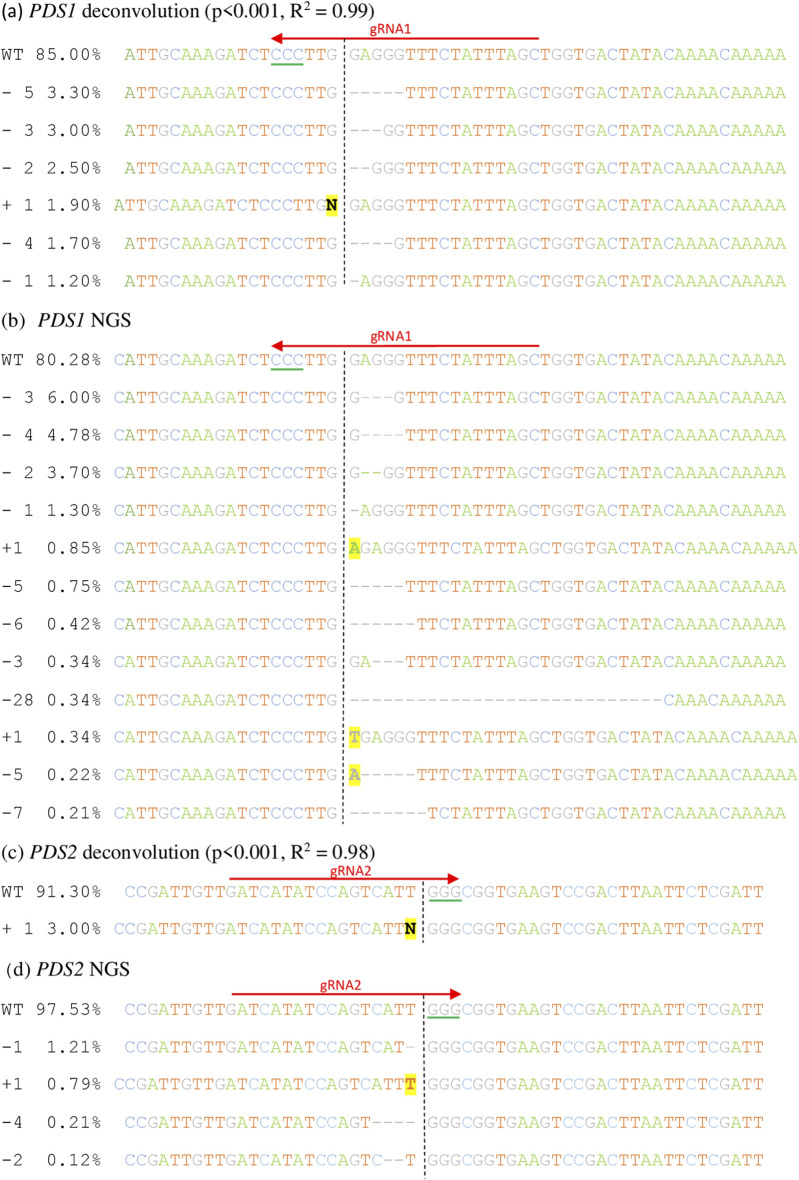
Estimated indels predicted by Sanger sequencing/TIDE deconvolution and actual indels detected by NGS. **(A,B)** *PDS1* indels **(C,D)** *PDS2* indels. N refers to estimated insertions of unknown nucleotides from TIDE analysis. Vertical dashed lines indicate gRNA cut sites, red arrows indicate gRNA sequences and green lines indicate PAM sequences. All sequences are shown in the 5′ to 3′ direction. Note that gRNA1 binds in the 3′ to 5′ direction.

Sanger sequencing followed by deconvolution algorithmically estimates genome editing efficiency by aligning the sequence trace files of a gene-edited sequence to a WT sequence. Using the gRNA sequence to determine the probable cut site, deconvolution programmes compare the individual basepair confidence of the two sequences to estimate what indels are present in the gene-edited sample and their frequencies (Brinkman et al., 2014; Bloh et al., 2021). TIDE deconvolution estimated editing efficiencies of 14% for *PDS1* with predicted indels ranging from +1 to −5 (Figure 3A). Despite the T7EI assay not producing any visible cleavage bands, editing efficiency was estimated at 6.2% for *PDS2* with the most common predicted indel of +1 (Figure 3C). Aberrant signal increased directly downstream of the gRNA binding site, particularly for *PDS1*, which was also visible on the chromatogram (Supplementary Figures S3A, C).

Amplicon sequencing followed by CRISPR analysis revealed 19.0% editing efficiency at *PDS1* with 12 variants (minimum presence in sequence population of 0.1%) ranging from −28 bp to +1 bp (Figure 3B). The most common indel was a 3 bp deletion present at 6% and there was some variation in where the DSB occurred. Some variation may be genuine, but for the most common mutations, DSB variance could be a sequencing artifact due to the start and end of the deletion both being a guanine nucleotide. *PDS2* had a 2.3% editing efficiency, with four variants (present at >0.1% of sequences) ranging from −4 bp to +1 bp (Figure 3D). The highest frequency indel was a 1 bp deletion present at 1.2%. CRISPR analysis of *PG*, WRKY52, *NPR1* revealed an editing efficiency of 1.2%, 0.3% and 6.0% respectively (Supplementary Figure S2).

## Discussion

These results represent the first instance of DNA-free RNP-mediated genome editing in raspberry protoplast. The transfection efficiency of 19% with pre-assembled CRISPR RNPs is substantial and in line with existing research; recent meta-analysis revealed that the average transfection efficiency of protoplast transfection with PEG was 17% (Rustgi et al., 2022). If gene edited protoplasts can be regenerated into raspberry plants, a 19% transfection efficiency would likely result in higher recovery rate of transformed plants than *Agrobacterium*-based methods in raspberry, which is currently between 0.5%–2% (Mathews et al., 1995; Georgieva et al., 2008). However, the editing efficiencies identified here are highly variable between different gRNAs (0.2%–19%), and while similar variation has been found elsewhere (Rustgi et al., 2022), more consistency in editing efficiency would be beneficial. Differences in gRNA efficiency is hypothesised to be due to variance in the accessibility of chromatin at the binding locus (Lee et al., 2024; Van Campenhout et al., 2019; Uusi-Mäkelä et al., 2018), therefore large-scale screening of gRNAs may be unavoidable. Nonetheless, successful DNA-free editing in *PG*, *WRKY52* and *NPR1* demonstrates the versatility of this protocol and its potential with agriculturally relevant functional targets. The development of novel protoplast-based transfection protocols in crop species has increased rapidly in recent years, with new methods in grapevine (Scintilla et al., 2022; Najafi et al., 2023; Tricoli and Debernardi, 2024), tomato (Lin et al., 2022; Liu et al., 2022), strawberry (Barceló et al., 2019; Gou et al., 2020), lettuce (Park et al., 2019), and potato (Moon et al., 2021), reflecting widespread interest. As genome-editing research accelerates in *Arabidopsis*, tobacco, wheat and rice (Shan et al., 2020), it will be vital to invest in method development in a wider range of crops such as raspberry to ensure that NGBTs can be fully deployed. With established DNA-free methods, raspberry breeders could target gene orthologs involved in firmness (e.g., *PG* (Atkinson et al., 2012; Posé et al., 2013)), *B. cinerea* resistance (e.g., *WRKY52* (Jia et al., 2020)*; NPR1* [Li et al., 2020; Li et al., 2021)], and powdery mildew resistance [e.g., *WRY52* (Wang et al., 2017)] that may improve shelf life, a major breeding target (Funt and Hall, 2013). Fruit flavour/taste, cane habit, plant architecture, aphid resistance and local climate adaptation (drought/flood tolerance) could also be broader targets for raspberry breeding through genome editing if associated genomic and phenomic links can be elucidated. This study demonstrates the amenability of raspberry to protoplast isolation, with considerably higher protoplast yields (average 1.5 × 10^7^ protoplast g^-1^) than previously reported (3.93 × 10^6^ g^-1^) (Phan et al., 1997). Yields from BWP102 are also higher than recent reports in woody species such as grapevine (6.6 × 10^6^ g^-1^) (Scintilla et al., 2022) and comparable to yields of 1 × 10^7^ protoplast g^-1^ seen in *Arabidopsis* (Yoo et al., 2007). The most important variable for successful protoplast isolation in raspberry, as found elsewhere (Yoo et al., 2007; Barceló et al., 2019), is the tissue quality used as a protoplast source. The outbreeding nature and high heterozygosity of raspberry adds complexity, compared to many other inbreeding species, as seeds cannot be used to grow sterile, soft, young plants for enzymolysis. As such, each raspberry seed is genetically different, and the genetic backgrounds of elite cultivars must be preserved to maintain berry consistency and quality. Therefore, vegetative stem cultures are a good alternative protoplast source. However, stem cultures are time and energy intensive, susceptible to contamination, and the health/quality of the canes is hugely influential on the protoplast yield. Although not systematically explored here, anecdotal evidence suggests indicators that improve protoplast yield include bright green stems that had deep red thorns and healthy lower leaves, alongside rapid growth of plantlets (within 10 days) from axillary buds in tissue culture. In future, isolation from callus could be an alternative protoplast source (Kang et al., 2020) to solve issues such as contamination and yield inconsistency.

Raspberry tissue remained mostly undigested despite 16 h of digestion, in contrast to *Arabidopsis* where leaves almost fully disintegrate into the enzymolysis solution (Jeong et al., 2021). As a woody species, raspberry likely has higher concentrations of structural polymers, such as lignin and cellulose, compared to model species. We hypothesise that levels of structural polymers in raspberry have a positive correlation to age and abiotic stress (Wang et al., 2016). Therefore, high levels of structural polymers may interfere with protoplast isolation (Brandt et al., 2020), resulting in lower yields from plantlets derived from older canes in poor health.

Pre-assembled RNPs were used in this study for several reasons. Primarily, non-transgenic crops are preferable as new legislation in England (Genetic Technology, 2023) permits only precision bred crops with mutations that could theoretically be produced by natural mutagenesis. Similar legislation is planned in the European Union (European Commission, 2021). The genomes of genetically modified crops inevitably contain foreign DNA (*Agrobacterium* T-DNA, Cas9/gRNA DNA or plasmid sequences) which need to be removed through backcrossing. As previously stated, backcrossing is not practical in raspberry, and in many other soft fruits, as the species is highly heterozygous and outbreeding. Public perception of transgenic food also remains a major issue (Woźniak-Gientka et al., 2022) and would likely influence marketability and eventual sales. Transgene-free cultivars generated by pre-assembled RNPs with enhanced phenotypes present a solution that research shows is more acceptable with consumers when used to improve sustainability or for societal benefit (Sprink et al., 2022). Furthermore, the ability to rapidly introduce targeted non-transgenic mutations directly in clones with elite genetic backgrounds would be a significant advantage compared to the years of selection and trials required for conventional raspberry breeding. Floricane cultivars of raspberry take 2 years to fruit, thus RNP-based methods represent a great increase in breeding speed and accuracy. However, the above advantages still rely upon the elucidation of a protoplast regeneration protocol in raspberry (Phan et al., 1997).

Plasmid-derived RNPs would likely be effective in this protocol, and we have successfully tested plasmid YFP expression in BWP102 protoplasts (Supplementary Figure S4). However, the risk of genomic plasmid integration (Kim et al., 2014) resulted in a preference for pre-assembled RNPs. Commercially synthesised Cas9 and gRNA enable high purity and sequence accuracy, which promotes better editing efficiencies. Furthermore, it reduces the technical knowledge required to construct new plasmids for transfection. However, synthetic gRNAs and Cas9 are expensive, therefore once gRNAs are validated, it may be cost-efficient for further work to synthesise RNPs in-house.

The cleavage test validated the efficacy of the gRNAs *in vitro*. Screening of gRNAs through cleavage tests is recommended prior to protoplast transfection (Brandt et al., 2020) to validate the activity of gRNAs using minimal components. Availability of cultivar-specific genome assemblies were crucial for the design of specific gRNAs and oligonucleotides with no sequence mismatches or off-target complementarity. The assembly used in this study is intended for publication (Kevei et al., unpub.), however other raspberry cultivar genome sequences are available (Davik et al., 2022; Price et al., 2023).

Quantifying genome editing efficiency in an amplicon pool is complex. T7EI and Sanger sequencing/deconvolution were used initially, which revealed positive results for *PDS1*, therefore amplicon sequencing was additionally conducted for comprehensive analysis. Sanger sequencing followed by computational deconvolution offers a low-cost alternative when NGS is financially unfeasible. Many free-to-use programs exist, namely, TIDE (Brinkman et al., 2014), ICE (Conant et al., 2022) and DECODR (Bloh et al., 2021). While useful, discrepancies between different programs mean they cannot be singularly relied upon (Brockman et al., 2023). Thus, if only using Sanger sequencing, editing estimates should be complemented with *in vitro* proof with T7EI digestion. Taken together, these two analysis techniques estimate what genotypes are present in the population and their relative frequencies at low cost. In this study, the T7EI assay demonstrated successful editing with RNP1, however did not validate the evidence of editing with RNP2 from TIDE, likely because editing was at a much lower frequency. The additional third band present in the T7EI *PDS1* sample could be explained by the endonuclease binding to a proportion of the DNA, reducing mobility through the agarose gel (New England Biolabs, 2024). Only amplicon sequencing was sensitive enough to detect the low editing efficiency of RNP2. Comparing the results of deconvolution and NGS reveals the former to be a rough estimation of true indels and their frequencies. Many correct indels were detected, however frequencies within the sequence pool were not accurate. Until recently, amplicon sequencing has been prohibitively expensive, and so methods such as T7EI and deconvolution were appealing. However, the decreasing cost of sequencing technologies, use of short-read (<500 bp) sequencing and the lack of high levels of accuracy with alternative methods, as identified here, indicates that amplicon NGS is the best choice for the detection of genome editing.

*PDS* was chosen as a proof-of-concept reporter gene as null mutants would present a distinctive albino phenotype early in protoplast regeneration. We also demonstrate editing in three agriculturally-relevant functional genes, null mutants of which have been shown to have increased firmness (*PG*) (Atkinson et al., 2012; Posé et al., 2013) and resistance to *B. cinerea* (*WRKY52* and *NPR1*) (Jia et al., 2020; Li et al., 2021) and powdery mildew (*WRKY52*) (Wang et al., 2017; Li et al., 2021) in other fruit species. In tandem with genome assemblies, this method enables rapid editing of any loci within the raspberry genome. However, as widely documented (Reed and Bargmann, 2021; Liu et al., 2022), protoplast regeneration of gene edited elite lines remains the greatest challenge to commercial implementation.

Reed and Bargmann (2021); Liu et al. (2022) This study aims to instigate further research into precision breeding in *Rubus* species by providing a straightforward, effective and reproducible protoplast isolation and quantifiable DNA-free genome editing protocol. Avenues have been opened for future work on gene expression and function. If protoplast regeneration methods can be elucidated in raspberry, genetic improvement of many raspberry traits will become feasible, with substantial benefits for the sustainability and efficiency of raspberry production.

## Data Availability

Datasets are available in a publicly accessible repository: https://www.ncbi.nlm.nih.gov/bioproject/?term=PRJNA1245325.
