# Supplementary material for: DNA-free CRISPR genome editing in raspberry (Rubus idaeus) protoplast through RNP-mediated transfection
Source: Front Genome Ed. 2025 Jun 30;7:1589431. doi: 10.3389/fgeed.2025.1589431 (PMC12256493; doi:10.3389/fgeed.2025.1589431)
Supplement: Supplementary file 2 [file Table1.docx]

Supplementary Information

**DNA-free CRISPR Genome Editing in Raspberry (*Rubus idaeus*) Protoplasts through RNP-mediated Protoplast Transfection**

Table S1: Table 1: Oligonucleotides and gRNAs used in this study for *PG, WRKY52* and *NPR1*. Predicted cleavage products were deduced assuming DSB 3bp upstream from PAM site. NGS primer pairs flank the same respective gRNA binding site. gRNA, guide RNA; RNP, ribonucleoprotein complex; bp, basepair; NGS, next generation sequencing*.*

| **399** | ***PG* CRISPR site** | ***WRKY52* CRISPR site** | ***NPR1* CRISPR site** |
| --- | --- | --- | --- |
| **Forward Primer**  **(5’ to 3’)** | CTCTAGTTTTCAGCGGGCCA | AGGAGTACCCTAAATATGACAGAAAC | TAGAAAGCCCTGCCTCAACA |
| **Reverse Primer**  **(5’ to 3’)** | GTGAGCGGGTTAAGGTCCAT | AGGGTCTCCATTTCCCAGGT | GGAATCTGAGGGGATACACTGC |
| **gRNA sequence**  **(5’ to 3’)** | GGAGGAACAGTTGATGCTCGCGG | GGAGATGTGGAGGGACCCAAGGG | GCACACGAAGAGGATATACAGGG |
| **Amplicon size (bp)** | 399 | 362 | 311 |
| **gRNA binding site within amplicon (bp)** | 166 | 270 | 127 |
| **Predicted cleavage products (bp)** | 166 + 233 | 92 + 270 | 127 + 184 |


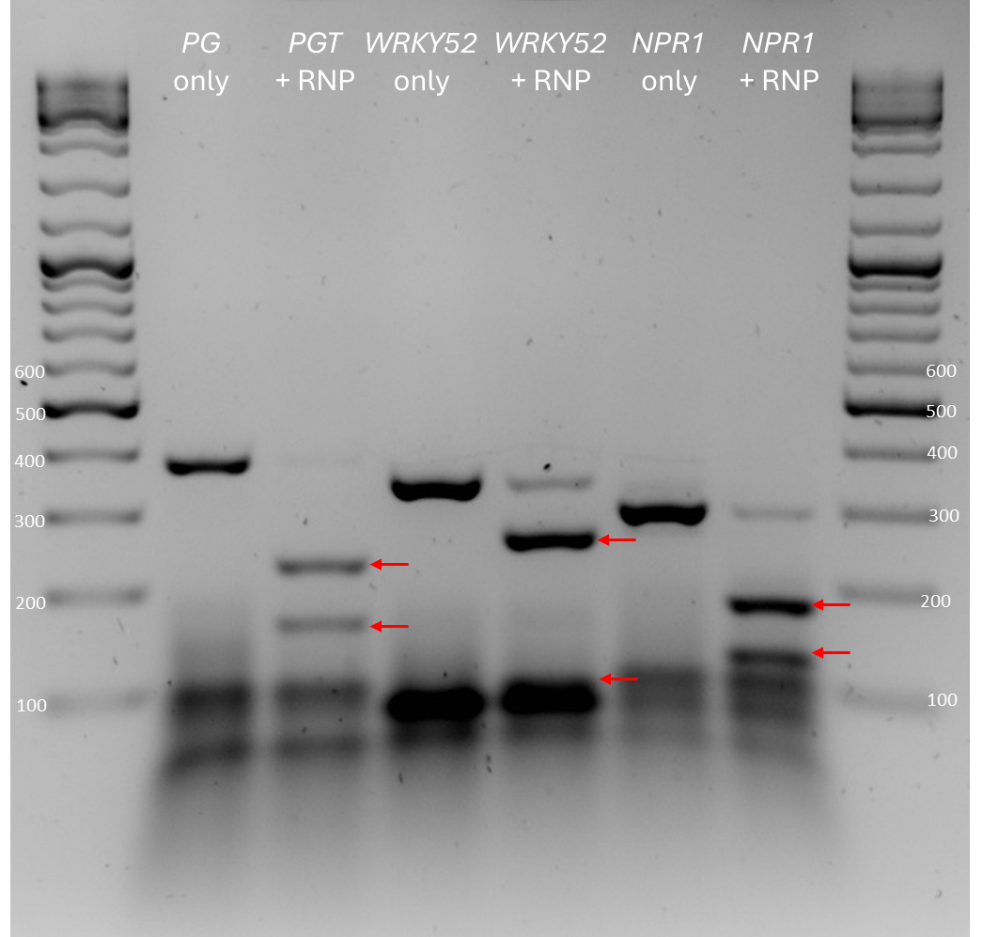
Figure S1: Detection of *in vitro* cleavage test RNP activity in leaf-derived PCR product of *PG, WRKY52* and *NPR1*. Red arrows indicate cleavage products that are of expected sizes and only found in RNP samples compared to Table S1. Note smearing at ~100bp and below caused by residual gRNA is found in both RNP and negative control samples.

Figure S2: Indels detected by Amplicon NGS of *PG, WRKY52* and *NPR1* gene edited protoplast-derived PCR products. Vertical dashed lines indicate gRNA cut sites, red arrows indicate gRNA binding sites and green lines indicate PAM sequences. All sequences are shown in the 5’ to 3’ direction. Font size for *WRKY52* gene edits has been reduced to include large 55bp insertion.

*PG* NGS

WT 98.35% AAGGTCACCGGCCTCTCTATCTACGGAGGAACAGTTGATGC TCGCGGAGCTGGATTT

-4 0.76% AAGGTCACCGGCCTCTCTATCTACGGAGGAACAGTTG---- TCGCGGAGCTGGATTT

-3 0.23% AAGGTCACCGGCCTCTCTATCTACGGAGGAACAGTTGAT-- -CGCGGAGCTGGATTT

*WKRY52* NGS

WT 99.72% AGATGTGGAGGGACC CAAGGGCAAAGGAGATGGATATCCACCGTCTGATTCGTGGAATTGGAGGAAGTACGGCCAAAAA

+55 0.17% AGACGTGGAGGGACC **ACCGTAACCCGGCTTCCGGTTCATCCCGCATCGCCAGTTCTGCTTACCAAAAATC**CAA…

+1 0.11% AGATGTGGAGGGACC **A**CAAGGGCAAAGGAGATGGATATCCACCGTCTGATTCGTGGAATTGGAGGAAGTACGGCCAAAA

*NPR1* NGS

WT 93.92% AGTAATTTTCCGGACAAGCACACGAAGAGGATAT ACAGGGCTTTGGATTCGGATGAT

-2 2.07% AGTAATTTTCCGGACAAGCACACGAAGAGGATA- -CAGGGCTTTGGATTCGGATGAT

-3 1.27% AGTAATTTTCCGGACAAGCACACGAAGAGG---T ACAGGGCTTTGGATTCGGATGAT

-4 1.14% AGTAATTTTCCGGACAAGCACACGAAGAGGA--- -CAGGGCTTTGGATTCGGATGAT

-1 0.63% AGTAATTTTCCGGACAAGCACACGAAGAGGAT-T ACAGGGCTTTGGATTCGGATGAT

-3 0.49% AGTAATTTTCCGGACAAGCACACGAAGAGGA--- ACAGGGCTTTGGATTCGGATGAT

-3 0.49% AGTAATTTTCCGGACAAGCACACGAAGAGGA--- ACAGGGCTTTGGATTCGGATGAT

+1 0.36% AGTAATTTTCCGGACAAGCACACGAAGAGGATAT **T**ACAGGGCACACGAAGAGGATGA


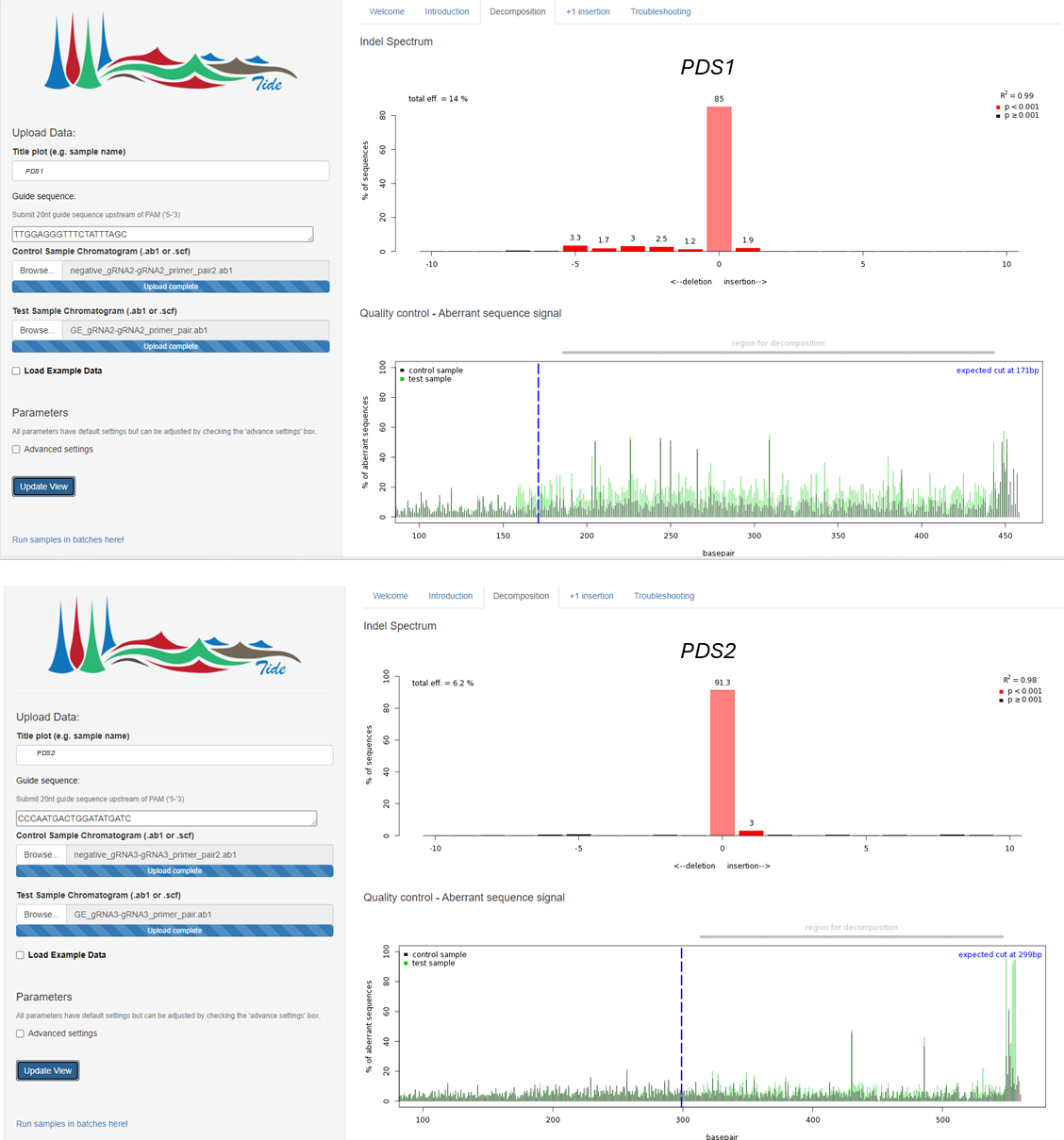
Figure S3: Screenshot of deconvolution result from TIDE of *PDS1* (a) and *PDS2* (b). Note increase in aberrant sequence signal after gRNA1 expected cut site. (c, d) screenshot of Sanger sequencing chromatogram in Geneious Prime for *PDS1* (c) and *PDS2* (d)*,* showing decrease in nucleotide consensus downstream of highlighted gRNA cut site in *PDS1* only.

(b)

(a)


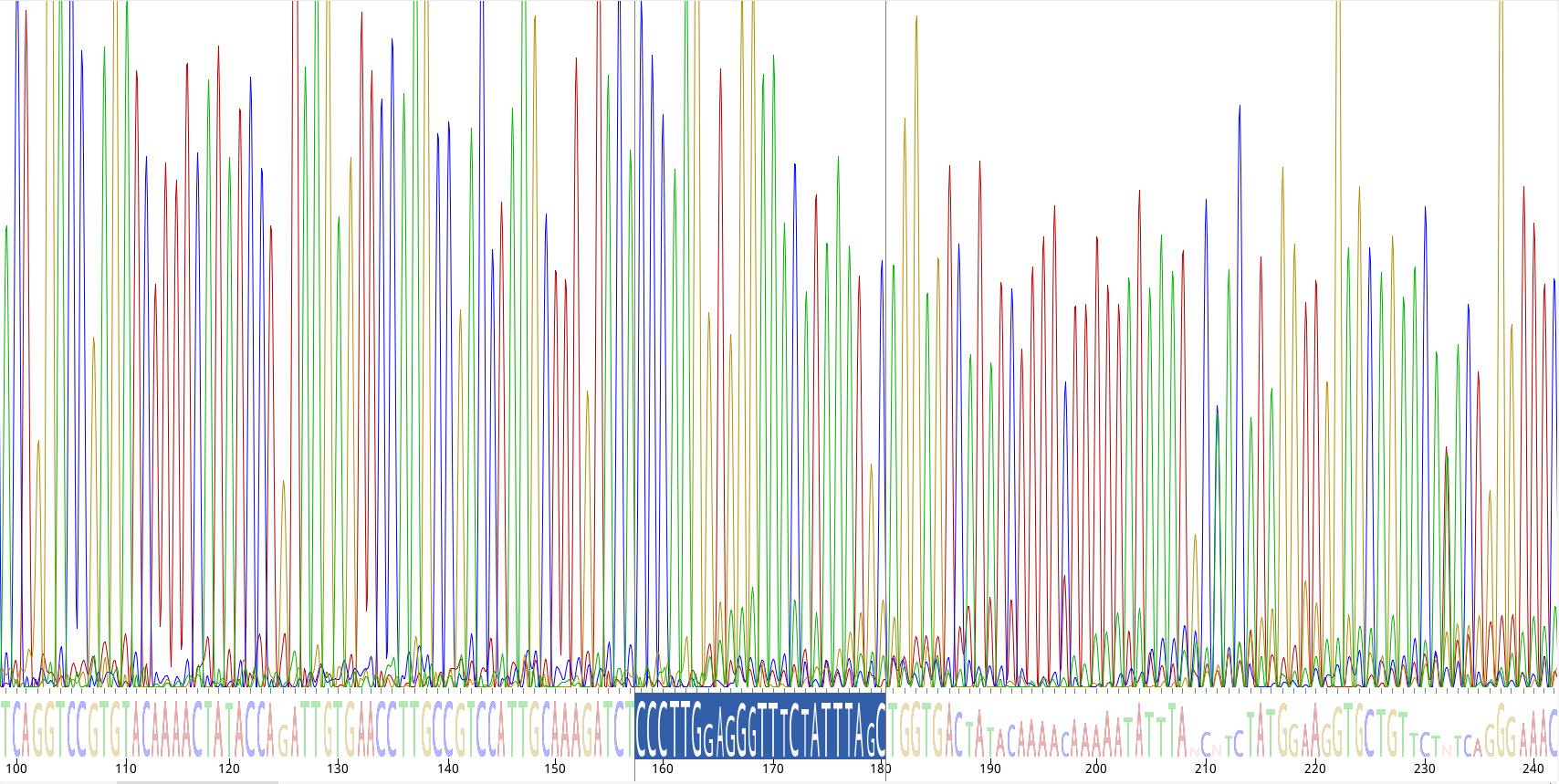

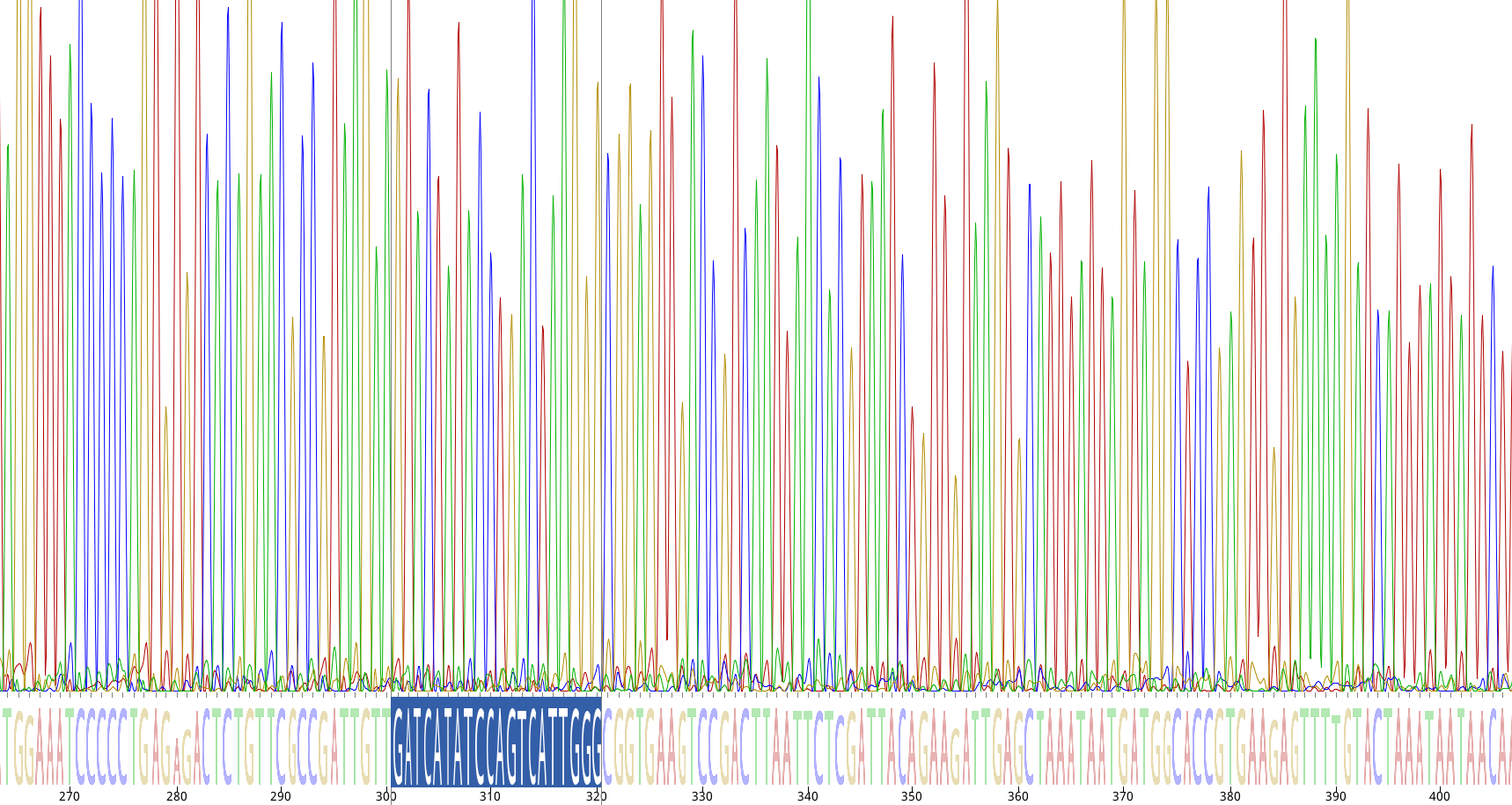


(d)

(c)

Figure S4: (a) Close up of BWP102 protoplast transfected with YFP plasmid; (b) YFP-containing plasmid used to transfect BWP102 protoplasts (c) 4x magnification image of sample of BWP102 protoplast showing widespread transfection. Error bar in (a) is 50µM and (c) is 200µM.

(b)

c)d)

(a)

c)d)


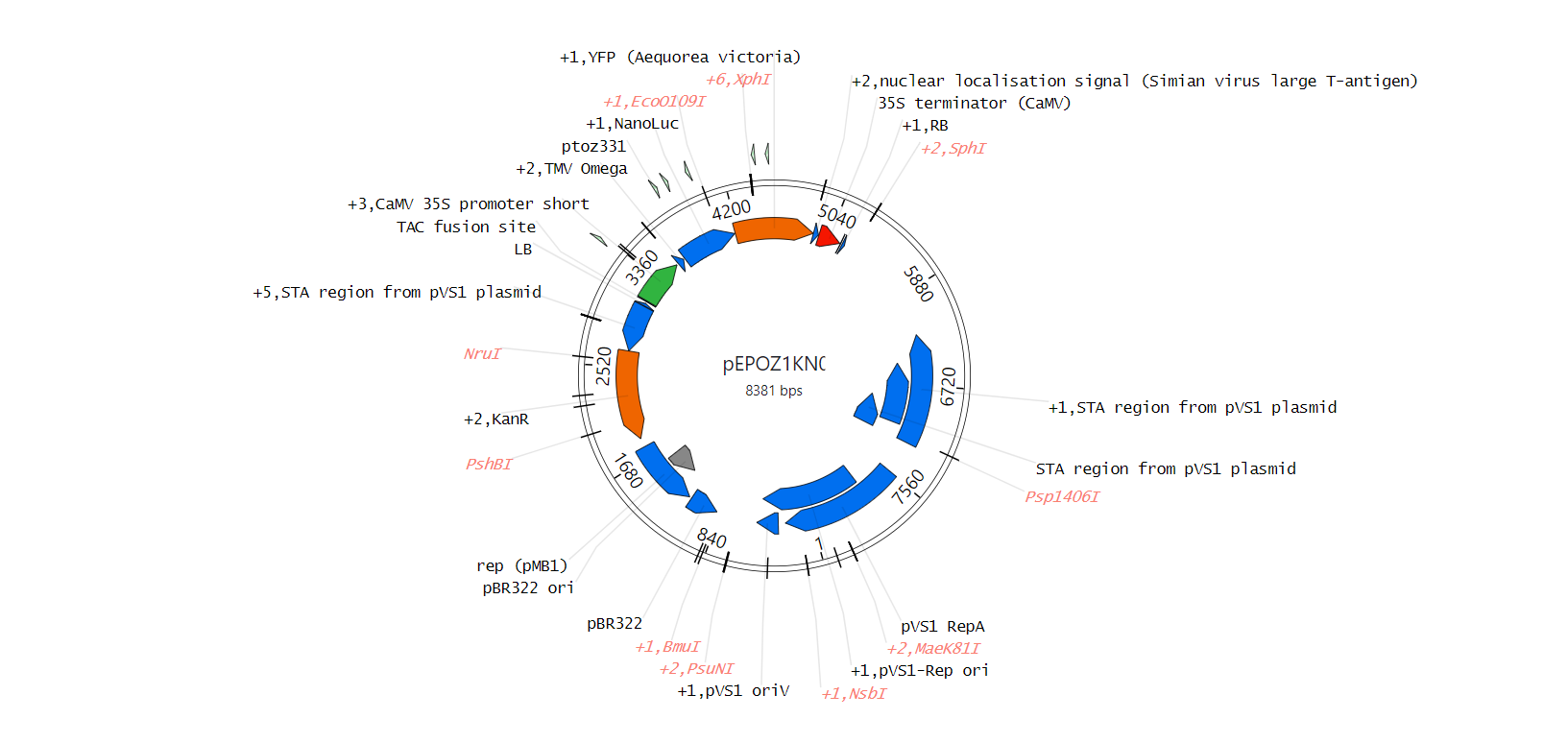

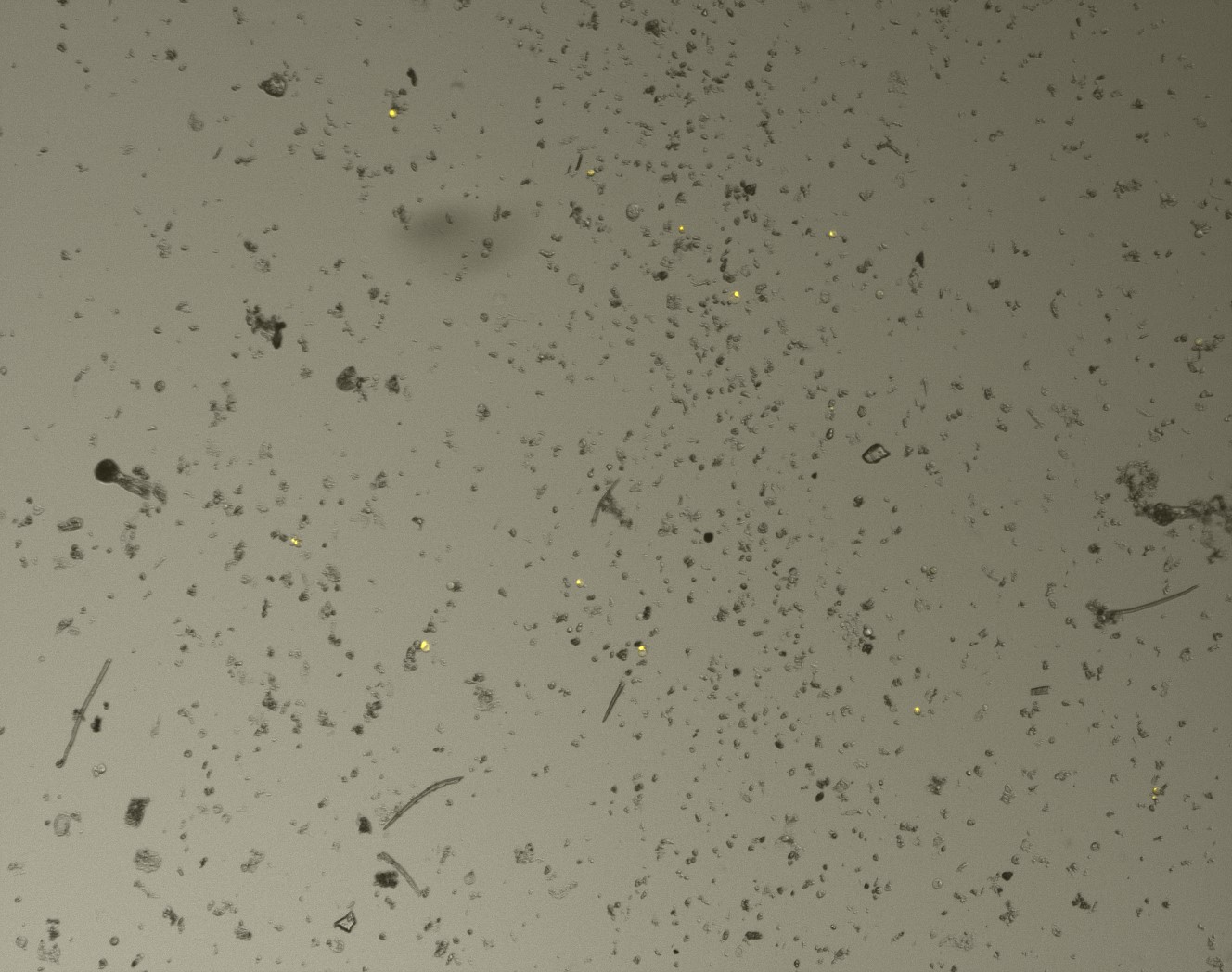

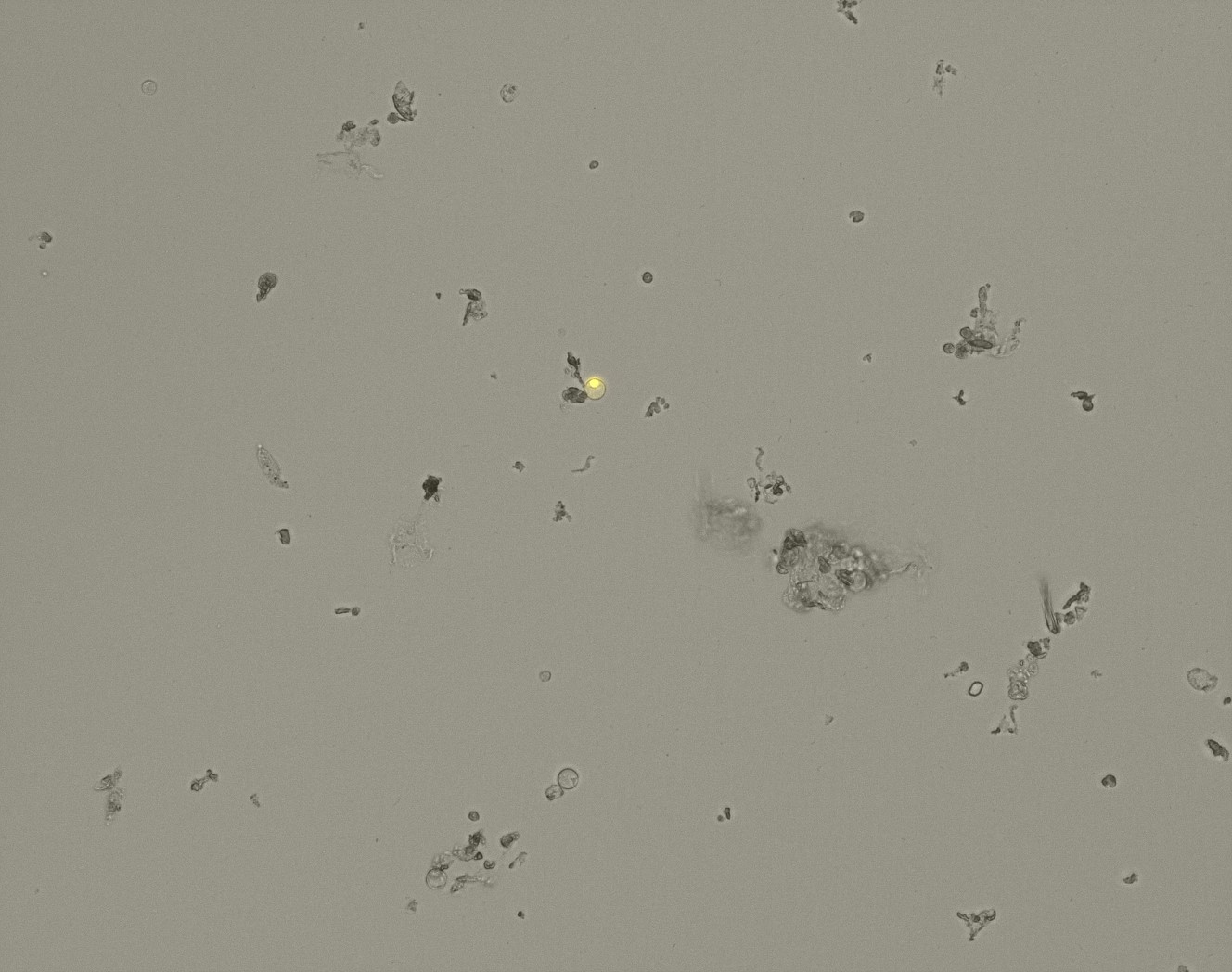


(c)

c)d)
